# Evaluation of pentoxifylline and ferrous sulfate for treatment of lower limb venous ulcers

**DOI:** 10.1590/1677-5449.200167

**Published:** 2021-05-24

**Authors:** Priscilla Cardoso Lemos, Esdras Marques Lins, Flavia Cristina Morone Pinto, José Lamartine de Andrade Aguiar, Fernanda Appolonio, Francisco Breno

**Affiliations:** 1 Universidade Federal de Pernambuco – UFPE, Recife, PE, Brasil.

**Keywords:** iron deficiency anemia, venous ulcer, venous insufficiency, ferrous sulfate, pentoxifylline

## Abstract

**Background:**

Venous ulcers (VU) are the most advanced stage of chronic venous disease (CVD) of the lower limbs. They are frequently associated with episodes of hemorrhage that can provoke chronic anemia (CA), delaying healing. There are no studies in the literature analyzing the prevalence of CA among patients with VU of the lower limbs and few studies have analyzed use of pentoxifylline to treat VU of the lower limbs.

**Objectives:**

To evaluate the prevalence of CA in patients with lower limb VU and responses to treatment with ferrous sulfate (SF) compared with a combination of SF plus pentoxifylline as adjuvant treatment for VU of the lower limbs.

**Methods:**

A total of 67 patients with lower limb VU were recruited from a Lymphedema and Angiodysplasia Clinic at the Hospital das Clínicas, Recife, PE, Brazil. After initial clinical and laboratory assessments, patients diagnosed with CA were randomized into one of two groups: a control group, given SF (900 mg/day oral route), or a study group, treated with SF (900 mg/day oral route) and pentoxifylline (1,200 mg/day). All were reassessed after 90 days.

**Results:**

Twenty-seven patients (40%) had CA. After treatment, increases were observed in hemoglobin and hematocrit levels, iron kinetics had improved, and both depth and area of VU had reduced in both groups, without statistically significant differences.

**Conclusions:**

A high prevalence of anemia was detected in the study population. The combination of SF and pentoxifylline was not more effective than SF alone for adjuvant treatment of VU of the lower limbs.

## INTRODUCTION

Venous ulcers (VU) are the most advanced stage of chronic venous disease (CVD) of the lower limbs. The majority of patients who have this condition also have low disposable income and, as a consequence, have unreliable access to health services. As a consequence, they seek medical attention when they already have extensive ulcers, which are often associated with chronic hemorrhage episodes, since in the majority of cases this type of wound develops in areas with varicose veins that rupture easily. These repeated episodes can provoke chronic anemia (CA), which, in turn, as has been well established in the literature, can delay or even prevent VU healing.^1^^-^ 8

In addition to standard treatment with dressing and elastic compression, there are now adjuvant measures proposed for treatment of VU of the lower limbs, including administration of medications such as pentoxifylline, which acts on red blood cell deformability. Published data indicate that use of this drug is associated with improved healing process, compared with placebo, when combined with elastic compression and with no other type of treatment.^9^^-^ 12 According to a systematic review from the Cochrane Database of Systematic Reviews,^9^ in the presence of concomitant compressive therapy, groups treated with pentoxifylline demonstrated a higher probability of healing compared with treatment with placebo. There are few studies that have adequately studied use of pentoxifylline to promote healing of this type of ulcer and there are no studies that have evaluated its use in combination with ferrous sulfate (SF) for this purpose.

In the light of the above, the objective of this study was to evaluate the prevalence of chronic anemia among patients with lower limb VU and their response to the treatment with SF compared to pentoxifylline and SF for adjuvant treatment of VU of the lower limbs.

## METHODOLOGY

A total of 67 patients with CVD and VU of the lower limbs were recruited from a Lymphedema and Angiodysplasia Clinic at the Hospital das Clínicas, Recife, PE, Brazil, and assessed from August 2015 to August 2016 (consecutive sample). The design employed was a prospective intervention study (clinical trial) with simple randomization. All participants signed free and informed consent forms after being given information about the study.

The sample was calculated on the basis of the expected frequency of active or healed VU in the population with CVD (3.6%), considering an acceptable margin of error of 5%, a 95% confidence interval (CI), and heterogeneity of 50%. This calculation was based on the normal distribution.

The following Formula 1 was used to calculate the sample size:

$$\text{n =}\frac{\text{Z}\times \text{Z}\left[\text{P(1 – P)}\right]}{(\text{D}\times \text{D)}}$$ (1)

Where “Z” represents the normal distribution — in this case, Z = 1.96 (95%CI); “P” is the expected prevalence; and “D” is the maximum acceptable estimation error (5%). The primary outcome was presence of CA and secondary outcomes were response to treatment of CA and lower limb VU at 90 days.

Study participants were adult patients with VU of the lower limbs and a laboratory diagnosis of CA. Diagnosis of CA was defined as: iron deficiency anemia secondary to chronic bleeding (hemoglobin < 12 g/dL); normal reticulocytes, ferritin, serum iron; transferrin saturation at the lower limits of the normal reference or below it; and total iron binding capacity (TIBC) at the upper limit of the reference range or elevated beyond it. Presence of peripheral pulses in the lower limbs was also an inclusion criterion.

Patients were excluded from the study if they had infected VU, or were diagnosed with VU of lower limbs and also had chronic gastrointestinal and/or genitourinary bleeding, iron-deficiency anemia, or anemia of other etiologies — such as thalassemias, and inflammatory, sideroblastic, aplastic, hemolytic (autoimmune and non immune), or megaloblastic anemias, considering patients’ prior histories and laboratory differential diagnosis.

Patients who had serum hemoglobin below 12 g/dL and hemometric and kinetic iron results compatible with iron deficiency anemia secondary to chronic bleeding were randomized into one of two treatment groups: a control group (G1) comprising 13 patients who were treated with the standard dressings by the vascular surgery service at the Hospital das Clínicas, Recife, PE, and oral administration of SF (300 mg, three times a day) for 90 days, or a study group (G2) comprising 14 patients treated with standard dressings and oral administration of both SF (300 mg, three times a day) and pentoxifylline (400 mg, three times a day), also for 90 days.

Clinical assessment of VU of the lower limbs was conducted according to the MEASURE methodology^13^ followed by analysis of blood tests. After 90 days of treatment, all patients underwent clinical reassessment with the MEASURE system and laboratory tests were repeated.

The data collected were organized in spreadsheets (Microsoft Office Excel^®^, New Mexico, United States) and analyzed using GraphPad Prism^®^ version 4.0 (GraphPad Software, California, United States). Analysis of variance (ANOVA) was used for continuous variables that fit a normal curve (parametric) which were compared using Student’s *t* test. The Kruskal-Wallis and Mann-Whitney U tests were used to analyze non-parametric data. Fisher’s exact test was used to analyze proportions and percentages.

The values from laboratory test results for the parameters (hemoglobin, hematocrit, reticulocytes, ferritin, serum iron, iron binding, and transferrin) were organized for multivariate analysis with ANOVA, followed by the *t* test for independent samples. The same variables were also analyzed for differences in each parameter tested before and after treatment, within each group (D90 – D0) and between groups (Group 2 – Group 1), in this case taking the laboratory reference values for each parameter. Analysis of differences was inferred using the Mann-Whitney U test.

Hemoglobin was defined as the primary diagnostic parameter for anemia. These data were first used to calculate descriptive statistics, and then the efficacy of treatment with SF combined with pentoxifylline was analyzed using the following formula: [Efficacy = % treatment failure in the SF group - % treatment failure in the SF + pentoxifylline group / % treatment failure in the SF group].^14^

Odds ratios (OR) were calculated to measure the likelihood that a person would achieve therapeutic success from anemia treatment using pentoxifylline combined with SF. The parameter defined as the cutoff value for normal hemoglobin was ≥12 g/dL.

The Miettinen formula was applied, for which the result is equivalent to the lower limit of the confidence interval (95%CI) of the OR, as follows: Strength of association = antilog {[1-1.96/√x^2^] .1n(OR)}, with 5% significance (i.e., Z = 1.96). If the result is greater than 1, the association is considered significant.

The nonparametric Friedman test was used for the parameters assessed with the MEASURE methodology because this test is calculated using the ranks of data rather than their numeric values. For all situations, the maximum probability of error acceptable for rejection of the null hypothesis was set at 5% (p < 0.05), considering a 95% safety margin.

The study was approved by the Human Research Ethics Committee at the Hospital das Clínicas, Universidade Federal de Pernambuco (UFPE), Recife, PE, Brazil, for data collection under protocol number 1.134.878.

## RESULTS

Twenty-seven of the 67 patients with lower limb VU had CA (40%). The mean age of the 67 patients assessed was 63 years (± standard deviation [SD] 11.78). Female patients accounted for 71% (n=47) of the sample. In the subset of 27 patients with lower limb VU and CA, mean age was 72.6 years (± SD 12.88) in G1 and 59.4 years (± SD 10.35) in G2, with a statistically significant difference between groups (p = 0.0072).

Comparison of data on the characteristics of the lower limb VU from the first consultation (D0) with those from the reassessment at 90 days (D90) showed that in G1 there was an increase in the number of patients with hyperpigmentation and a decrease in the number of patients with lipodermatosclerosis, both without statistical difference (p = 1.000); an increase in granulation tissue in the VU bed, but without statistical difference (p = 0.9853); an increase in the number of patients with VU exudate, but without statistical difference (p = 1.000); and absence of fetid odor at both assessments and reduction in the depth of VU, but without statistically significant difference (p = 0.8566 in the right lower limb [RLL] and p = 0.6522 in the left lower limb [LLL]). The was also a reduction in the area of ulcers, but without statistically significant difference (p = 0.9097 for the RLL and p = 0.9583 for the LLL).

Analysis of the characteristics of the lower limb VU in G2 comparing D0 to D90 revealed no changes in hyperpigmentation or lipodermatosclerosis; reduction in granulation tissue in the VU bed (p = 0.9853), absence of fetid odor, infection, or exudate at both assessments; reduction in the depth of ulcers, but without statistically significant difference (p = 0.8566 for RLL and p = 0.6522 for LLL); and reduction in the area of ulcers, also without statistically significant difference (p = 0.9097 for the RLL and p = 0.9583 for the LLL) (Table 1).

**Table 1:** Characteristics of lower limb VU in anemic patients.

**VARIABLES**	**Group 1**		**Group 2**		*p*-value
	**Ferrous sulfate**		**Ferrous sulfate + pentoxifylline**		
	**D0**	**D90**	**D0**	**D90**	
**Ulcer characteristics (%)**					
Hyperpigmentation	61.54	66.67	57.14	57.14	1.0000
Lipodermatosclerosis	38.46	33.33	42.86	42.86	
**Tissue types (%)**					
Granulation	76.92	83.33	71.43	64.29	0.9853
Granulation + fibrin	15.38	16.67	28.57	35.71	
Fibrin	7.69	0.00	0.00	0.00	
**Infected (%)**					
Yes	0.00	8.33	0.00	0.00	1.0000
No	100.00	91.67	100.00	100.00	
**Exudate (%)**					
Present	0.00	8.33	0.00	0.00	1.0000
Absent	100.00	91.67	100.00	100.00	
**Odor (%)**					
Fetid	0.00	0.00	0.00	0.00	__
Sui generis	100.00	100.00	100.00	100.00	
**Depth (cm)**					
Right					
0	37.50	62.50	0.00	18.18	0.8566
< 0.3	50.00	12.50	54.55	72.73	
0.3-0.6	12.50	25.00	27.27	0.00	
0.7-1.0	0.00	0.00	9.09	0.00	
1.1-2.0	0.00	0.00	0.00	0.00	
Left					
0	22.22	37.50	0.00	0.00	0.6522
< 0.3	44.44	37.50	60.00	60.00	
0.3-0.6	11.11	25.00	40.00	40.00	
0.7-1.0	11.11	0.00	0.00	0.00	
1.1-2.0	11.11	0.00	0.00	0.00	
**Area (cm^2^)**					
Right					
0.5-5.0	25.00	50.00	18.18	54.55	0.9097
5.0-10.0	25.00	12.50	27.27	9.09	
> 10.1	50.00	37.50	54.55	9.09	
Left					
0.5-5.0	33.33	62.50	20.00	40.00	0.9583
5.0-10.0	22.22	0.00	20.00	0.00	
> 10.1	44.44	37.50	60.00	60.00	

Values expressed as percentage (%). The parameters “infected”, “exudate”, and “odor” were analyzed using descriptive statistics. The Friedman test was used. If p < 0.05. D0 = data from first consultation; D90 = data from 90-day reassessment; VU = venous ulcer.

Analysis of laboratory test results from before (D0) and after (D90) CA treatment in G1 and G2 revealed increases in hemoglobin, hematocrit, reticulocytes, ferritin, transferrin, and serum iron levels; and a reduction in total iron binding capacity. There was also a statistically significant improvement in anemia after 90 days of treatment in G2, in which treatment was with SF and pentoxifylline (p = 0.0262).

Analysis of the efficacy of anemia treatment revealed improved parameters in both groups, with a greater likelihood of cure of anemia in G2 than G1 (OR 2.5, 95%CI 0.51-12.30), but without statistical power (p = 0.4312).

Considering treatment of lower limb VU and CA, there were improvements in hemoglobin levels and reductions in ulcer depth, reflected in the healing process in 4 (33%) patients in the control group, compared to 8 (57%) patients in the study group, with a higher likelihood of improvement in the study group (OR 2.7, 95%CI 0.5380-13.22). There was no statistically significant difference between groups (p = 0.2671). The analysis of ulcer depth was defined as the parameter for evaluation of healing after treatment (D90). The data on ulcer area were not considered, because they were not representative (Tables 2 and 3).

**Table 2:** Distribution of patients who exhibited increased hemoglobin (Hb) levels and reduction in ulcer depth, in control and study groups.

	**If ulcer depth < 0.3 (cm) and Hb ≥ 12 (g/dL)** *	
**G2**		
**Ferrous sulfate + pentoxifylline**		
	Depth < 0.3	Hb ≥ 12
Yes	13	8
**G1**		
**Ferrous sulfate**		
	Depth < 0.3	Hb ≥ 12
Yes	9	4

* Patients had Hb < 12 before treatment.

**Table 3:** Analysis of treatment efficacy according to increase in hemoglobin (Hb) level and reduction in ulcer depth in control and study groups.

Efficacy analysis	If Hb ≥ 12 (g/dL) and ulcer depth < 0.3 (cm)				OR	95%CI	Efficacy (%)	p Value
	Yes		No					
Factor	Actual	Expected	Actual	Expected				
**G2**								
**Ferrous sulfate + pentoxifylline**	8	57.1	6	42.9	2.7	0.5380-13.22	35.7	0.2671
**G1**								
**Ferrous sulfate**	4	33.3	8	66.7				

RR = (a/a+b) / (c/c+d). RR = 1.71. There is a higher “risk” of increase in Hb level and reduction in ulcer depth in group taking pentoxifylline + ferrous sulfate. OR = (a*d) / (b*c). 2.7x greater chance of not curing anemia and depth when treated with only with pentoxifylline. Efficacy = 1-RR. If the RR is greater than 1 (as in the example), then there is a higher risk in the exposed group relative to the not exposed group. Fisher’s exact test was used to analyze the data, in a contingency table. RR = relative risk; OR = odds ratio.

## DISCUSSION

Laboratory diagnosis of CA in this study employed laboratory tests and criteria that are well-established in the literature. The method employed to assess VU of the lower limbs was set out in the MEASURE protocol, is widely employed in the routines of other hospitals and clinics, and has been frequently used in scientific studies of cutaneous ulcers.^13^^,^ 15 ^-^ 17

The treatment for CA consisted of iron replacement. The SF administration employed, at an oral dosage of 900 mg/day, is the current recommendation in the literature for treatment of cases of iron deficiency anemia secondary to chronic episodes of bleeding.^14^^,^ 18 ^-^ 20

Laboratory diagnosis of CA revealed mean hemoglobin levels in the mild anemia range and in the same way the iron kinetics parameters (trend for reduced ferritin, transferrin, and serum iron and increased TIBC) in almost half of the patients assessed in this study. These laboratory test results are compatible with iron deficiency anemia by progressive blood loss (anemia due to chronic bleeding).^21^^,^ 22

Recent Brazilian data show a 9% prevalence of iron deficiency anemia among elderly people. The present study detected a much higher prevalence (40%) of iron deficiency anemia (secondary to chronic bleeding of VU). In 1970, J. Marks and A. Shuster stated that anemia and cutaneous ulcers were so common that it would be expected to find them both in the same patient and that, in some cases, this would occur because of a direct relationship. Despite this, the authors of this study did not specifically investigate the association between VU of the lower limbs and CA.^23^^-^ 25

With regard to treatment of CA, improvement in anemia was observed in both groups at the end of the 90-day treatment, with a statistically significant difference in the study group, which was treated with SF and pentoxifylline. There are reports in the literature on the action of pentoxifylline in treatment of anemia in inflammatory disease associated with iron deficiency anemia secondary to chronic blood loss (mixed anemia), such as anemia of chronic renal failure,^26^ but there are no consistent reports related to the action of pentoxifylline for treatment or purely iron-deficiency anemia due to chronic bleeding.^27^

At the end of 90 days, in both treatment groups it was observed that there were reductions in both depth and area of ulcers and that the majority of wounds had granulation tissue, reflecting improved transcutaneous oxygen supply and an adequate wound healing process. However, these reductions were not statistically significant when assessment times were compared (at diagnosis and after treatment) in each group or between groups, even though some studies in the literature had shown a significant positive effect of pentoxifylline on healing of venous ulcers.^9^^-^ 12 These results are similar to those found by authors who have reported that pentoxifylline is a satisfactory adjuvant medication for healing of VU of lower limbs, but the data do not prove its efficacy.^28^^,^ 29

It is extremely important to identify patients with lower limb VU associated with CA, in order to indicate the need for simultaneous treatment of CA, contributing to faster ulcer healing, improving patient quality of life, and reducing expenditure on dressings.

## LIMITATIONS OF THE STUDY

The sample calculation was based on the expected VU prevalence at the tertiary University hospital where the study was conducted. In this case, the expected prevalence of VU was less than in the general population, since the Lymphedema and Angiodysplasia Clinic at this hospital has a larger number of patients with arterial diseases and patients who need vascular access for hemodialysis, rather than those with CVD of the lower limbs. As a result, the sample size was smaller than it would have been if the prevalence of VU in the general population had been used.

## CONCLUSIONS

A high prevalence of anemia was detected in the study population. The combination of SF with pentoxifylline did not show greater efficacy than use of SF alone for adjuvant treatment of VU of the lower limbs.
