# Supplementary material for: Evaluation of pentoxifylline and ferrous sulfate for treatment of lower limb venous ulcers
Source: J Vasc Bras. 2021 May 24;20:e2020167. doi: 10.1590/1677-5449.200167 (PMC8153203; doi:10.1590/1677-5449.200167)
Supplement: Translation

# Translation: Avaliação da pentoxifilina e do sulfato ferroso no tratamento da úlcera varicosa dos membros inferiores

**Authors:** Priscilla Cardoso Lemos, Esdras Marques Lins, Flavia Cristina Morone Pinto, José Lamartine de Andrade Aguiar, Fernanda Appolonio, Francisco Breno

**Affiliations:** 1 Universidade Federal de Pernambuco – UFPE, Recife, PE, Brasil.

**Keywords:** anemia ferropriva, úlcera varicosa, insuficiência venosa, sulfato ferroso, pentoxifilina

## Abstract

**Contexto:**

A úlcera varicosa (UV) é o estágio mais avançado da doença venosa crônica (DVC) dos membros inferiores (MMII), frequentemente associada a episódios de hemorragia que podem provocar anemia crônica (AC) e retardar a sua cicatrização. Não há, na literatura, trabalhos que avaliem a prevalência da AC nos portadores de UV dos MMII, e poucos trabalhos analisam o uso da pentoxifilina no tratamento das UV dos MMII.

**Objetivos:**

Avaliar a prevalência da AC nos pacientes portadores de UV de MMII e a resposta terapêutica ao sulfato ferroso (SF) e a associação da pentoxifilina com SF no tratamento adjuvante das UV dos MMII.

**Métodos:**

Foram avaliados 67 pacientes portadores de UV de MMII atendidos no ambulatório de Cirurgia Vascular do Hospital das Clínicas, Recife, PE. Após as avaliações clínica e laboratorial iniciais, os pacientes diagnosticados com AC foram randomizados em dois grupos: o grupo controle, que recebeu SF (900 mg/dia via oral), e o grupo de estudo, tratado com SF (900 mg/dia via oral) e pentoxifilina (1.200 mg/dia). Todos foram reavaliados após 90 dias.

**Resultados:**

Entre os pacientes avaliados, 27 (40%) apresentavam AC. Após o tratamento, foram observados aumento dos níveis de hemoglobina e de hematócrito e melhora das taxas da cinética do ferro, assim como a diminuição da profundidade e da área das UV em ambos os grupos, sem diferença estatística.

**Conclusões:**

Foi encontrada alta prevalência de anemia na população estudada. A associação do SF com a pentoxifilina não se mostrou mais eficaz do que o emprego isolado do SF no tratamento adjuvante da UV dos MMII.

## INTRODUÇÃO

A úlcera varicosa (UV) representa o estágio mais avançado da doença venosa crônica (DVC) dos membros inferiores (MMII). A maioria dos pacientes portadores dessa enfermidade apresenta baixo poder aquisitivo e, consequentemente, tem acesso precário aos serviços de saúde. Devido a isso, procuram o atendimento médico quando apresentam úlceras extensas, que são frequentemente associadas a episódios crônicos de hemorragia, já que esse tipo de ferida, na maioria dos casos, se desenvolve sobre uma área contendo veias varicosas que facilmente sofrem ruptura. Esses episódios repetidos podem provocar anemia crônica (AC), a qual, por sua vez, como já bem demonstrado na literatura, pode retardar ou até impedir a cicatrização da UV^1^^-^ 8.

Além do tratamento padrão com curativos e elastocompressão, existem medidas adjuvantes atualmente propostas para o tratamento das UV dos MMII, como o uso de medicamentos como a pentoxifilina, que atua sobre a flexibilidade das hemácias. Dados da literatura apontam para a melhora do processo cicatricial com o uso dessa droga, comparando-a com placebo, associada à elastocompressão e a nenhum tipo de tratamento^9^^-^ 12. Segundo a revisão sistemática da *Cochrane Database of Systematic Reviews* 9, na presença concomitante de terapêutica compressiva, o grupo de tratamento com pentoxifilina demonstrou maior probabilidade de cicatrização em comparação ao tratamento com placebo. São escassos os trabalhos que estudam adequadamente o uso da pentoxifilina na cicatrização desse tipo de ferida, e não existem estudos avaliando sua associação com o sulfato ferroso (SF) para essa finalidade.

A partir dessas considerações, o objetivo deste estudo foi avaliar a prevalência da anemia crônica nos pacientes portadores de UV de MMII e a resposta terapêutica ao SF comparada à associação da pentoxifilina e SF no tratamento adjuvante das UV dos MMII.

## METODOLOGIA

Foram avaliados 67 pacientes portadores de DVC e UV dos MMII, provenientes do Ambulatório de Cirurgia Vascular do Hospital das Clínicas, Recife, PE, no período entre agosto de 2015 e agosto de 2016 (amostra consecutiva). O modelo de estudo empregado foi de intervenção (ensaio clínico), prospectivo e randomizado simples. Todos os participantes assinaram o termo de consentimento livre e esclarecido após receberem informações sobre o estudo.

A amostra foi calculada a partir da frequência esperada (3,6%) de UV ativa ou cicatrizada na população com DVC, considerando a margem de erro aceitável de 5%, o intervalo de confiança (IC) de 95% e o nível de heterogeneidade de 50%. O cálculo foi baseado em uma distribuição normal.

Para o cálculo da amostra, foi utilizada a seguinte Fórmula 1:

$$\text{n =}\frac{\text{Z}\times \text{Z}\left[\text{P(1 – P)}\right]}{(\text{D}\times \text{D)}}$$ (1)

Na qual “Z” se refere à distribuição normal — neste caso, Z = 1,96 (IC 95%); “P” é a prevalência esperada; e “D”, o erro máximo aceitável na estimativa (5%). Foi considerado como desfecho primário a presença de AC e, como secundário, a resposta ao tratamento das UV de MMII e da AC após 90 dias.

Foram incluídos no estudo pacientes adultos, portadores de UV dos MMII, com diagnóstico laboratorial de AC. Foi considerado diagnóstico de AC: a anemia ferropriva por sangramento crônico (hemoglobina < 12 g/dL), reticulócitos normais, ferritina, ferro sérico e saturação da transferrina no limite inferior da referência ou diminuídos e, por fim, capacidade total de ligação do ferro (TIBC) no limite superior da referência ou aumentada. A presença de pulsos periféricos nos MMII também foi critério de inclusão.

Foram excluídos do estudo os pacientes que apresentavam UV infectada e os pacientes diagnosticados com UV de MMII que apresentavam sangramento crônico gastrointestinal e/ou gênito-urinário, anemia carencial, assim como anemias de outras etiologias — a exemplo as inflamatórias, talassemias, sideroblásticas, aplásicas, hemolíticas (autoimune e não imune) e megaloblásticas, considerando história prévia do paciente e diagnóstico laboratorial diferencial.

Os pacientes que apresentaram hemoglobina sérica menor que 12 g/dL, índices hematimétricos e cinética do ferro compatíveis com anemia ferropriva por sangramento crônico foram distribuídos de forma randomizada em dois grupos de tratamento: o grupo controle G1 – composto por 13 pacientes, que receberam tratamento através de curativo padrão do Serviço de Cirurgia Vascular do Hospital das Clínicas, Recife, PE, acrescido da administração oral de SF (300 mg, três vezes ao dia) por 90 dias — e o grupo de estudo G2 – composto por 14 pacientes, tratados através de curativo padrão e da administração oral de SF (300 mg, três vezes ao dia) e pentoxifilina (400 mg, três vezes ao dia), também por 90 dias.

A avaliação clínica da UV dos MMII foi realizada conforme a metodologia MEASURE^13^ seguida da análise dos exames sanguíneos. Todos os pacientes foram submetidos a reavaliação clínica com aplicação do sistema MEASURE e novos exames laboratoriais após 90 dias de tratamento.

Os dados obtidos foram organizados em planilhas (Microsoft Office Excel^®^, Novo México, EUA) e analisados pelo programa *GraphPad Prism* ^®^ versão 4.0 (GraphPad Software, Califórnia, EUA). A análise de variância (ANOVA) foi usada para tratamento das variáveis contínuas que se ajustavam a uma curva normal (paramétricas) e foram comparadas através do teste *t* de Student. Os testes de Kruskal-Wallis e U de Mann-Whitney foram usados para a análise de dados não-paramétricos. Para a análise de proporções e porcentagens, utilizou-se o teste Exato de Fisher.

Para os valores referentes aos exames laboratoriais, os parâmetros (hemoglobina, hematócrito, reticulócitos, ferritina, ferro sérico, ligação com o ferro e transferrina) foram organizados para análise multivariada, pela ANOVA, seguida do teste *t* não-pareado. Para as mesmas variáveis, foi realizada também a diferença dos valores laboratoriais de cada parâmetro antes e depois do tratamento, dentro de cada grupo (D90 – D0) e entre os grupos (Grupo 2 – Grupo 1) – nesse caso, respeitando-se os valores de referência laboratoriais de cada parâmetro. A análise das diferenças foi inferida pelo teste U de Mann-Whitney.

A hemoglobina foi definida como parâmetro de diagnóstico principal para a anemia. Para esses dados, foi aplicada estatística descritiva, seguida da avaliação da eficácia do tratamento com a associação de pentoxifilina, através da fórmula: [Eficácia = % falha terapêutica no grupo SF - % falha terapêutica no grupo SF + pentoxifilina / % falha terapêutica no grupo SF]^14^.

O cálculo da *odds ratio* [razão de chances (OR)] foi realizado para mensurar as chances de uma pessoa obter sucesso terapêutico para o tratamento da anemia, com o uso da pentoxifilina associada ao SF. Foi considerado como parâmetro o valor de normalidade da hemoglobina maior ou igual a 12 g/dL.

Realizada a aplicação da fórmula de Miettinen, cujo resultado é representado pelo limite inferior do intervalo de confiança (IC95%) da OR: Força de associação = antilog {[1-1,96/√x^2^] .1n(OR)}, com 5% de significância, ou seja, Z = 1,96. Se o resultado for maior que 1, considera-se a associação significante.

Para os parâmetros avaliados através da metodologia MEASURE, foi aplicado o teste não paramétrico de Friedman, pois esse teste utiliza os *ranks* dos dados ao invés de seus valores brutos para o cálculo. Em todas as situações, a probabilidade máxima de erro aceitável para rejeição da hipótese nula foi de cinco % (p < 0,05), considerando a margem de segurança de 95%.

O estudo foi aprovado pelo Comitê de Ética e Pesquisa em Seres Humanos da Hospital das Clínicas, Universidade Federal de Pernambuco (UFPE), Recife, PE, para a coleta de dados sob o parecer 1.134.878.

## RESULTADOS

Entre os 67 pacientes portadores de UV dos MMII, 27 (40%) apresentavam AC. A média de idade dos 67 pacientes avaliados foi de 63 anos [± desvio padrão (DP) 11,78]. As pacientes do gênero feminino representavam 47 (71%) do total de pacientes avaliados. Quando considerados os 27 pacientes portadores de UV de MMII e AC, a idade média dos pacientes foi 72,6 anos (± DP 12,88) no G1 e 59,4 anos (± DP 10,35) no G2, havendo diferença estatística significante entre eles (p = 0,0072).

Em relação às características da UV dos MMII, quando comparados dados da primeira consulta (D0) e da reavaliação após 90 dias (D90), foi observado no G1 um aumento do número de pacientes que apresentavam hiperpigmentação e uma redução do número de pacientes que apresentavam lipodermatoesclerose, ambos sem diferença estatística (p = 1,000); um aumento do tecido de granulação no leito da UV, mas sem diferença estatística (p = 0,9853); um aumento do número de pacientes que apresentavam exsudato da UV, porém sem diferença estatística (p = 1,000); a ausência de odor fétido em ambos os momentos, a diminuição da profundidade da UV, porém sem diferença estatística significativa [p = 0,8566 no membro inferior direito (MID) e p = 0,6522 no membro inferior esquerdo (MIE)]. Foi observada ainda uma diminuição da área das lesões, mas sem diferença estatisticamente significativa (p = 0,9097 no MID e p = 0,9583 no MIE).

Quanto às características da UV de MMII no G2 comparadas na D0 e D90, foi observado que não houve alterações em relação à hiperpigmentação e à lipodermatoesclerose; ocorreu diminuição do tecido de granulação nos leitos das UV (p = 0,9853), ausência de odor fétido, infecção e exsudato em ambos os momentos; diminuição da profundidade das feridas, porém sem diferença estatística significativa (p = 0,8566 no MID e p = 0,6522 no MIE) e diminuição da área das feridas, também sem diferença estatisticamente significativa (p = 0,9097 no MID e p = 0,9583 no MIE) (Tabela 1).

**Tabela 1:** Características das UV dos MMII dos pacientes anêmicos.

**VARIÁVEIS**	**Grupo 1**		**Grupo 2**		p valor
	**Sulfato ferroso**		**Sulfato ferroso e pentoxifilina**		
	**D0**	**D90**	**D0**	**D90**	
**Características da úlcera (%)**					
Hiperpigmentação	61,54	66,67	57,14	57,14	1,0000
Lipodermatoesclerose	38,46	33,33	42,86	42,86	
**Tipo de tecido (%)**					
Granulação	76,92	83,33	71,43	64,29	0,9853
Granulação + fibrina	15,38	16,67	28,57	35,71	
Fibrina	7,69	0,00	0,00	0,00	
**Infectada (%)**					
Sim	0,00	8,33	0,00	0,00	1,0000
Não	100,00	91,67	100,00	100,00	
**Exsudato (%)**					
Presente	0,00	8,33	0,00	0,00	1,0000
Ausente	100,00	91,67	100,00	100,00	
**Odor (%)**					
Fétido	0,00	0,00	0,00	0,00	__
*Sui generis*	100,00	100,00	100,00	100,00	
**Profundidade (cm)**					
Direito					
0	37,50	62,50	0,00	18,18	0,8566
< 0,3	50,00	12,50	54,55	72,73	
0,3-0,6	12,50	25,00	27,27	0,00	
0,7-1,0	0,00	0,00	9,09	0,00	
1,1-2,0	0,00	0,00	0,00	0,00	
Esquerdo					
0	22,22	37,50	0,00	0,00	0,6522
< 0,3	44,44	37,50	60,00	60,00	
0,3-0,6	11,11	25,00	40,00	40,00	
0,7-1,0	11,11	0,00	0,00	0,00	
1,1-2,0	11,11	0,00	0,00	0,00	
**Área (cm^2^)**					
Direito					
0,5-5,0	25,00	50,00	18,18	54,55	0,9097
5,0-10,0	25,00	12,50	27,27	9,09	
> 10,1	50,00	37,50	54,55	9,09	
Esquerdo					
0,5-5,0	33,33	62,50	20,00	40,00	0,9583
5,0-10,0	22,22	0,00	20,00	0,00	
> 10,1	44,44	37,50	60,00	60,00	

Valores representados em porcentagem (%). Os parâmetros “infectada”, “exsudato” e “odor” foram avaliados por estatística descritiva. Aplicado teste de Friedman. Se p < 0,05. D0 = dados da primeira consulta; D90 = dados da reavaliação após 90 dias; MMII = membros inferiores; UV = úlcera varicosa.

Em relação aos exames laboratoriais, quando avaliados antes (D0) e após (D90) o tratamento da AC, no G1 e no G2, observou-se um aumento dos níveis de hemoglobina, hematócrito, reticulócitos, ferritina, transferrina e ferro sérico; além de um decréscimo da taxa de capacidade total de ligação ao ferro. Foi encontrada melhora da anemia estatisticamente significante após 90 dias de tratamento no G2, cuja terapêutica foi SF e pentoxifilina (p = 0,0262).

Com relação à eficácia do tratamento da anemia, houve melhora dos parâmetros em ambos os grupos, com uma chance maior de cura da anemia no G2 que no G1 (OR 2,5, IC 95% 0,51-12,30), porém sem força estatística (p = 0,4312).

Considerando o tratamento da UV de MMII e da AC, houve melhora dos níveis de hemoglobina e diminuição da profundidade das lesões, refletido no processo cicatricial da úlcera em 4 (33%) pacientes no grupo controle, ao passo que, no grupo estudo, 8 (57%) pacientes foram beneficiados, com uma chance maior de melhora no grupo estudo (OR 2,7, IC 95% 0,5380-13,22). Não houve diferença estatística significativa entre os grupos (p = 0,2671). A análise da profundidade foi considerada parâmetro da cicatrização das feridas avaliadas após o tratamento (D90). Os dados da área das úlceras não foram considerados, pois não foram expressivos (Tabelas 2 e 3).

**Tabela 2:** Disposição dos pacientes que obtiveram aumento da taxa de hemoglobina (Hb) e redução da profundidade da lesão, nos grupos controle e estudo.

	**Se profundidade da lesão < 0,3 (cm) e Hb ≥ 12 (g/dL)** ^*^	
**G2**		
**Sulfato ferroso e pentoxifilina**		
	Profundidade < 0,3	Hb ≥ 12
Sim	13	8
**G1**		
**Sulfato ferroso**		
	Profundidade < 0,3	Hb ≥ 12
Sim	9	4

* Considerando que não teve Hb ≥ 12 antes do tratamento.

**Tabela 3:** Análise da eficácia do tratamento pelo aumento da taxa de hemoglobina (Hb) e redução da profundidade da lesão, entre os grupos controle e estudo.

**Análise de eficácia**	**Se Hb ≥ 12 (g/dL) e profundidade da lesão < 0,3 (cm)**				OR	IC 95%	Eficácia (%)	p Valor
	**Sim**		**Não**					
Fator	Real	Esperado	Real	Esperado				
**G2**								
**Sulfato ferroso e pentoxifilina**	8	57,1	6	42,9	2,7	0,5380-13,22	35,7	0,2671
**G1**								
**Sulfato ferroso**	4	33,3	8	66,7				

RR = (a/a+b) / (c/c+d). RR = 1,71. Há uma maior chance de aumentar a taxa de Hb e diminuir a profundidade da UV com o uso de pentoxifilina + sulfato ferroso. OR = (a*d) / (b*c). 2,7x mais chances de não curar a anemia e profundidade quando tratado só com pentoxifilina. Eficácia = 1-RR. Se o RR é maior que 1 (como no exemplo), então há um maior risco do grupo exposto relativo ao grupo não exposto. Foi aplicado o teste Exato de Fisher para avaliação dos dados, tabulados em tabela de contingência. RR = risco relativo; OR = *odds ratio*.

## DISCUSSÃO

O diagnóstico laboratorial da AC neste estudo utilizou exames laboratoriais e critérios já bem estabelecidos na literatura. O método utilizado para avaliação das UV de MMII foi o estabelecido no protocolo MEASURE, que é amplamente executado na rotina de outros hospitais e ambulatórios e tem sido empregado frequentemente em trabalhos científicos que estudam as úlceras cutâneas^13^^,^ 15 ^-^ 17.

O tratamento da AC consistiu na reposição do ferro. A administração do SF empregada, na dose oral de 900 mg/dia, é atualmente recomendada pela literatura para o tratamento dos casos de anemia ferropriva secundária a episódios de sangramento crônico^14^^,^ 18 ^-^ 20.

O diagnóstico laboratorial da AC evidenciou níveis médios de hemoglobina correspondentes a faixa de anemia leve assim como as taxas da cinética do ferro (tendência para diminuição da ferritina, transferrina, ferro sérico e para aumento da TIBC) presente em quase metade dos pacientes avaliados no corrente estudo. Os achados laboratoriais são compatíveis com anemia ferropriva, por perda sanguínea progressiva (anemia por sangramento crônico)^21^^,^ 22.

Dados nacionais recentes demonstraram uma prevalência da anemia por deficiência de ferro entre idosos de 9%. O presente estudo demonstrou uma prevalência bem mais alta (40%) de anemia ferropriva (secundária a sangramento crônico da UV). Desde 1970, J. Marks e A. Shuster afirmavam que anemia e lesões cutâneas eram tão comuns que seria esperado encontrá-las juntas no mesmo paciente e, em alguns casos, ocorreria por relação direta. Apesar disso, os autores deste estudo não avaliaram especificamente a associação entre UV dos MMII e a AC^23^^-^ 25.

Com relação ao tratamento da AC, foi observado que, em ambos os grupos, ao fim dos 90 dias, houve melhora da anemia, com diferença estatisticamente significante no grupo estudo, cujo tratamento utilizou SF e pentoxifilina. Há relatos na literatura sobre a ação da pentoxifilina no tratamento da anemia de doença inflamatória associada à anemia ferropriva por perda sanguínea crônica (anemia mista), como a anemia da insuficiência renal crônica^26^, mas não existem relatos consistentes relativos à ação da pentoxifilina no tratamento da anemia puramente ferropriva por sangramento crônico^27^.

Foi observado, em ambos os grupos de tratamento, ao fim de 90 dias, que houve uma redução tanto da profundidade quanto da área das úlceras e a maioria das feridas apresentava tecido de granulação, refletindo melhora do aporte transcutâneo do oxigênio e adequado processo de cicatrização das feridas. No entanto, essa redução não teve diferença estatisticamente significativa quando comparados os momentos de avaliação (diagnóstico e após o tratamento) em cada grupo e entre os grupos, apesar de alguns estudos da literatura demonstrarem um efeito positivo significativo da pentoxifilina na cicatrização de úlceras varicosa^9^^-^ 12. Esses resultados são semelhantes aos encontrados por autores que relataram que a pentoxifilina é uma medicação adjuvante satisfatória na cicatrização das UV de MMII, mas os dados não conseguiram provar a sua eficácia^28^^,^ 29.

É de extrema importância identificar os pacientes portadores de UV de MMII associada a AC, a fim de indicar a necessidade de tratamento simultâneo da última condição e, assim, contribuir para a cicatrização mais rápida das lesões, melhorando tanto a qualidade de vida do paciente quanto os custos com a realização de curativos.

## LIMITAÇÕES DO ESTUDO

Para o cálculo da amostra, foi utilizada considerada a prevalência esperada da UV no hospital terciário universitário onde ocorreu a pesquisa. Nesse caso, a prevalência esperada da UV foi menor que a da população geral, já que no ambulatório de cirurgia vascular desse hospital há maior número de pacientes atendidos por doenças arteriais e de pacientes que precisam de acesso vascular para hemodiálise, em vez de portadores de DVC dos MMII. Nesse caso, o tamanho da amostra foi menor do que seria se fosse utilizada a prevalência da UV na população geral.

## CONCLUSÃO

Foi encontrada alta prevalência de anemia na população estudada. A associação do SF e a pentoxifilina não se mostrou mais eficaz do que o emprego isolado do SF no tratamento adjuvante da UV dos MMII.
